# Identifying dominant-negative actions of a dopamine transporter variant in patients with parkinsonism and neuropsychiatric disease

**DOI:** 10.1172/jci.insight.151496

**Published:** 2021-09-22

**Authors:** Freja Herborg, Kathrine L. Jensen, Sasha Tolstoy, Natascha V. Arends, Leonie P. Posselt, Aparna Shekar, Jenny I. Aguilar, Viktor K. Lund, Kevin Erreger, Mattias Rickhag, Matthew D. Lycas, Markus N. Lonsdale, Troels Rahbek-Clemmensen, Andreas T. Sørensen, Amy H. Newman, Annemette Løkkegaard, Ole Kjærulff, Thomas Werge, for the iPSYCH researchers, Lisbeth B. Møller, Heinrich J.G. Matthies, Aurelio Galli, Lena E. Hjermind, Ulrik Gether

**Affiliations:** 1 Molecular Neuropharmacology and Genetics Laboratory, Department of Neuroscience, Faculty of Health and Medical Sciences, University of Copenhagen, Copenhagen, Denmark.; 2 Department of Molecular Physiology and Biophysics, Vanderbilt University, Nashville, Tennessee, USA.; 3 Department of Clinical Physiology and Nuclear Medicine, Bispebjerg Hospital, Copenhagen, Denmark.; 4 National Institute on Drug Abuse Intramural Research Program, NIH, Baltimore, Maryland, USA.; 5 Department of Neurology, Bispebjerg Hospital, Copenhagen University Hospital, Copenhagen, Denmark.; 6 Institute of Biological Psychiatry, Mental Health Services Copenhagen, Copenhagen, Denmark.; 7 Department of Clinical Medicine, University of Copenhagen, Copenhagen, Denmark.; 8 Lundbeck Foundation Initiative for Integrative Psychiatric Research (iPSYCH), Copenhagen, Denmark.; 9 The iPSYCH researchers are detailed in Acknowledgments.; 10 Center for Applied Human Genetics, Kennedy Center, Copenhagen University Hospital, Glostrup, Denmark.; 11 Department of Surgery, University of Alabama, Birmingham, Alabama, USA.; 12 Danish Dementia Research Centre, Clinic of Neurogenetics, Department of Neurology, Rigshospitalet, Copenhagen University Hospital, Copenhagen, Denmark.; 13 Department of Cellular and Molecular Medicine, Section of Neurogenetics, Faculty of Health and Medical Sciences, University of Copenhagen, Copenhagen, Denmark.

**Keywords:** Cell Biology, Neuroscience, Molecular genetics, Parkinson disease, Psychiatric diseases

## Abstract

Dysfunctional dopaminergic neurotransmission is central to movement disorders and mental diseases. The dopamine transporter (DAT) regulates extracellular dopamine levels, but the genetic and mechanistic link between DAT function and dopamine-related pathologies is not clear. Particularly, the pathophysiological significance of monoallelic missense mutations in DAT is unknown. Here, we use clinical information, neuroimaging, and large-scale exome-sequencing data to uncover the occurrence and phenotypic spectrum of a DAT coding variant, DAT-K619N, which localizes to the critical C-terminal PSD-95/Discs-large/ZO-1 homology–binding motif of human DAT (hDAT). We identified the rare but recurrent hDAT-K619N variant in exome-sequenced samples of patients with neuropsychiatric diseases and a patient with early-onset neurodegenerative parkinsonism and comorbid neuropsychiatric disease. In cell cultures, hDAT-K619N displayed reduced uptake capacity, decreased surface expression, and accelerated turnover. Unilateral expression in mouse nigrostriatal neurons revealed differential effects of hDAT-K619N and hDAT-WT on dopamine-directed behaviors, and hDAT-K619N expression in *Drosophila* led to impairments in dopamine transmission with accompanying hyperlocomotion and age-dependent disturbances of the negative geotactic response. Moreover, cellular studies and viral expression of hDAT-K619N in mice demonstrated a dominant-negative effect of the hDAT-K619N mutant. Summarized, our results suggest that hDAT-K619N can effectuate dopamine dysfunction of pathological relevance in a dominant-negative manner.

## Graphical abstract

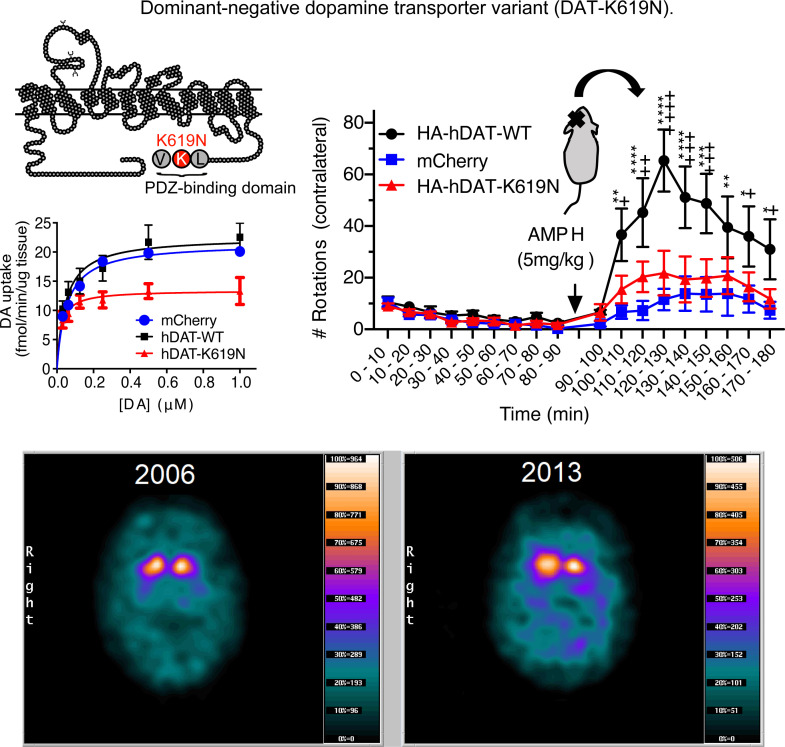

## Introduction

Dopamine (DA) exerts strong effects on human behavior by supporting motor initiation and exploration and through modulation of higher cognitive functions, such as reinforcement learning and motivation (1, 2). Disturbances in dopaminergic neurotransmission are widely implicated in neurological diseases, such as Parkinson disease, and in psychiatric disorders, including attention deficit hyperactivity disorder (ADHD), schizophrenia, bipolar disorder, and depression (1, 3–6). As a critical regulator of dopaminergic neurotransmission (7), the human DA transporter (hDAT) gene, *SLC6A3*, has received enduring attention in candidate gene studies of Parkinson disease and psychiatric diseases (8). DAT mediates high-affinity reuptake of DA and thereby regulates the spatiotemporal propagation of DA transients and ensures a synthesis-independent DA source to dopaminergic terminals (9). The important role of DAT in exerting behaviorally relevant control of DA homeostasis is substantiated by the acute and long-term effects of psychostimulant therapeutics and recreational drugs, such as amphetamine, methylphenidate, and cocaine, which all target DAT.

Although candidate gene studies of Parkinson disease and mental disorders have reported associations with *SLC6A3* (8, 10, 11), genome-wide association studies (GWAS) have not confirmed the existence of disease-associated common *SLC6A3* variants (12). However, several lines of evidence suggest that rare variants of larger effect size may link DAT dysfunction to parkinsonism and neuropsychiatric disease. Most importantly, a causal relationship between DAT dysfunction and human disease was established with the description of DAT deficiency syndrome (DTDS), in which inactivating mutations in *SLC6A3* give rise to a recessively inherited form of infantile parkinsonism dystonia (13). Classical DTDS presents in early infancy (14, 15); however, later studies have described cases with childhood/adolescent disease onset (16), and we recently identified an adult patient with compound heterozygote missense mutations in *SLC6A3* that only partially disrupt DAT function; this patient has an atypical form of DTDS with adult-onset of motor symptoms and comorbid neuropsychiatric disease (17). The identification of this patient adds to several reports of heterozygous carriers of coding variants in *SLC6A3* who have been diagnosed with neuropsychiatric disease, including bipolar disorder, ADHD, and autism spectrum disorder (ASD; refs. 18–24). Most of these DAT variants show deficits in DAT properties and/or trafficking and several cause behavioral changes in vivo, at least when homozygously expressed (17–20, 23–28). Moreover, *SLC6A3* shows a high degree of conservation and is constrained against loss-of-function variants — supporting the notion that perturbations of DAT function may contribute to malfunctioning states that impose the negative selection against such variants (29).

Here, we investigated the mechanistic and functional impact of a recurring rare coding hDAT variant, hDAT-K619N, on dopaminergic neurotransmission. We first present 2 unrelated patients who carry the hDAT-K619N variant. The first patient is an adult male with early-onset parkinsonism and comorbid neuropsychiatric disease, for whom we used single-photon emission CT (SPECT) scans to reveal a progressive loss of DAT availability. The second patient was diagnosed with ASD and identified in the Simons Simplex Collection of families with ASD. Importantly, the hDAT-K619N mutation is located in a C-terminal PSD-95/Discs-large/ZO-1 homology (PDZ) binding sequence, which is critical for DAT surface expression in vitro (30) and for striatal expression and surface stability in vivo (31). We showed that the hDAT-K619N variant was subject to accelerated cellular turnover that in turn caused impaired surface expression and reduced DA uptake capacity. This functional impairment was dominant-negative toward the WT transporter, as shown by in vitro experiments and viral overexpression in mice. In mice, we also found that unilateral overexpression of hDAT-WT and hDAT-K619N in nigrostriatal neurons caused diverging effects on DA-directed behavior and, by expressing the hDAT-K619N variant in *Drosophila*, we demonstrated that the mutant can drive dopaminergic dysfunction with associated alterations in locomotion. Finally, we used a large population-based exome-sequencing data set from patients with psychiatric disorders to estimate effect sizes, to our knowledge for the first time, for a rare DAT coding variant. We identified the hDAT-K619N mutation in an additional 21 patients, as well as in 5 control subjects, with a nominally significant association with bipolar disorder. Collectively, our study substantiates the putative role of rare coding hDAT variants as genetic risk factors in neuropsychiatric disease and lends further support to a possible link between genetic insult to DAT function and neurodegenerative parkinsonism in adults.

## Results

### Identification of the hDAT-K619N variant in 2 unrelated patients with early-onset parkinsonism and/or neuropsychiatric disease.

**Figure 1:**
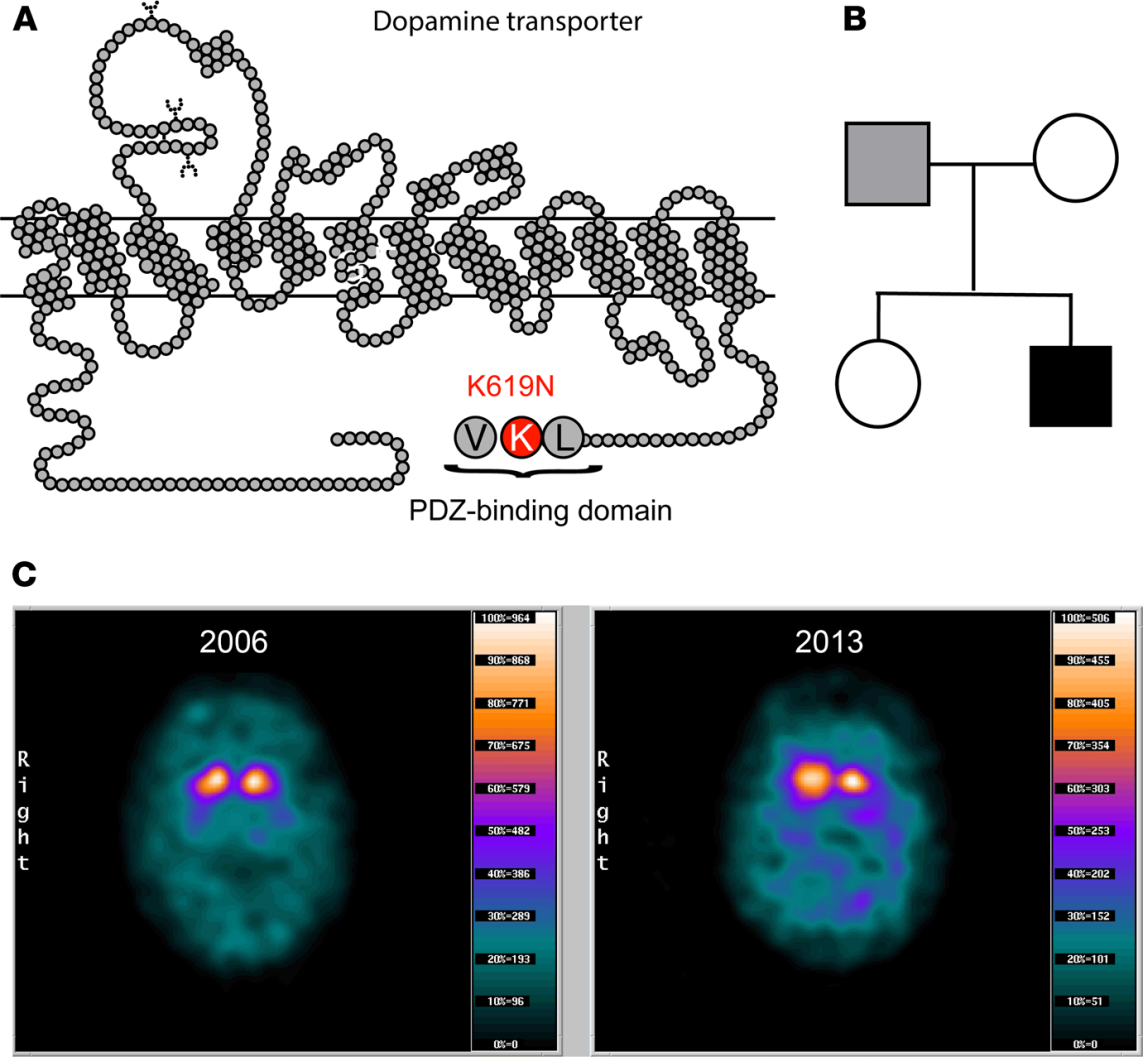
Identification of DAT-K619N in a patient with atypical parkinsonism and comorbid psychiatric disease. (**A**) Snake diagram of DAT demonstrating the C-terminal location of the DAT-K619N mutation within a PDZ-binding domain. (**B**) Family tree with the index patient 1 shown in black shading. Only the patient’s parents were available for genetic analysis, which revealed paternal transmission of the DAT-K619N allele. It is unknown whether the father has neurological or psychological symptoms. (**C**) [^123^I]FP-CIT SPECT imaging of patient 1 carrying the DAT-K619N variant. Two [^123^I]FP-CIT SPECT scans, acquired 7 years apart, suggest progressive neurodegeneration. Images were taken with identical procedures on the same scanner at age 34 (in 2006, left) and age 43 (in 2013, right). Quantification of DAT availability is presented in Table 1.****

In 2 unrelated male patients from independent samples, we identified the same single nucleotide substitution (c.1857G>C) in exon 15 of *SLC6A3*, giving rise to the coding variant hDAT-K619N. Interestingly, the K619 residue is part of a PDZ-binding sequence that has been reported to regulate DAT trafficking and to be essential for normal expression of DAT at striatal release sites (refs. 30–32 and Figure 1A). The first patient was identified in a previously described cohort of 91 patients with symptoms of early-onset parkinsonism, dystonia, or other unclassified movement disorders (17). He is a white male (aged 41 years at the time of referral) diagnosed with early-onset parkinsonism and neuropsychiatric disease. The second patient belonged to the Simons Simplex Collection (consisting of more than 2000 families with ASD) and was diagnosed with ASD (33). It is not known whether patient 2 has any movement-related comorbidity or not. Only patient 1 was available for further clinical evaluation. Inheritance investigations showed that the hDAT-K619N allele was paternally transmitted to patient 1 (Figure 1B), but it is not known whether the father has neurological or neuropsychiatric symptoms or signs because he did not want to be examined. According to clinical records, patient 1 reported insidious onset of right-sided hand tremor in his late thirties. His symptoms were initially interpreted as part of a psychiatric disorder and autism was suspected, but he was diagnosed with early-onset parkinsonism when his motor symptoms worsened. At age 41, he presented with unequivocal hemiparkinsonism with right-sided symptoms. A brain MRI scan at age 39 was reportedly normal, except for the presence of more small (2–4 mm) white matter lesions than expected for this age. Electroencephalography, as well as visual and motor-evoked potentials, were normal. Genetic testing for spinocerebellar ataxias, Huntington disease–like syndromes, and hereditary parkinsonism uncovered no extended trinucleotide repeat in *ATXN1-3*, *CACNA1A*, *TBP*, and *FMR1*; no pathogenic variants in *PRKN* and *GCH1*; and no hotspot variant in *LRRK2* *(*G2019S) or common point variants and copy number variations in *DJ1*, *PINK1*, *UCHL1*, *SNCA*, and *ATP13A2*. Further, no pathogenic variants were found in *NPC1*, *NPC2*, or *NOTCH3*. Patient 1 was not formally assessed by specialists in child psychiatry during childhood/adolescence. As an adult, however, he has had several episodes of self-injury and has been in intermittent contact with inpatient and outpatient psychiatric facilities. He was diagnosed with an unspecified personality disorder with evasive and schizophrenic traits (ICD-10-CM code F60.9) and periodic depression (F33.9) at age 43. Treatment of parkinsonian symptoms was initially attempted with DA agonists in low to moderate doses (with some positive effects but also side effects), and levodopa/carbidopa was later administered with a positive effect. Moreover, the patient has received treatments to improve his psychiatric symptoms, including risperidone in a low dose (discontinued because of side effects) and escitalopram for a shorter period. All treatments had variable compliance.

Patient 1 is currently 53 years old and presents with general bradykinesia, severe rigidity, and resting and intention tremor of the upper extremities without consistent sidedness of symptoms (Supplemental Video 1; supplemental material available online with this article; https://doi.org/10.1172/jci.insight.151496DS1). The tremor is slightly more irregular than the usual parkinsonian tremor, probably because of dystonic elements. His gait is parkinsonian with small shuffling steps, kyphosis, and severe difficulties in turning around (Supplemental Video 2). Postural instability is obvious, and a retropulsion pull test was not possible because he tends to fall spontaneously. He has severe hypomimia (Supplemental Video 3), but his eye movements show normal pursuit with complete range and no square wave jerks. Saccades are normal, including initiation, velocity, and range. His voice is slightly hoarse but not severely hypophonic (Supplemental Video 2). Current treatment is Sinemet (levodopa and carbidopa) (900 mg/d). He has been offered but declined advanced treatment (continued duodenal infusion of levodopa/carbidopa, DuoDopa).

### DAT-SPECT scans support progressive neurodegeneration.

The mechanisms through which genetic insult to DAT function causes either classical infantile DTDS or atypical DTDS with adult disease onset and psychiatric comorbidity are largely unknown. Interestingly, a DAT-SPECT scan of patient 1 from 2006 demonstrated a clear reduction in DAT binding in striatum (caudate nucleus and putamen) with the largest reduction seen on the left hemisphere, consistent with a right-sided predominance of the patient’s symptoms. Although the scan resembles those observed in neurodegenerative parkinsonism (34), the loss of DAT availability could, in principle, reflect both dopaminergic cell loss and reduced DAT expression connected to the hDAT-K619N variant. However, by comparing with a second DAT-SPECT scan acquired 7 years later, we identified an accelerated loss of [^123^I]-FP-CIT binding compared with the expected decline from age-matched controls (Figure 1C and Table 1). This suggests that loss of DA neurons is contributing to the loss of DAT binding and thus that dopaminergic neurodegeneration is part of the neuropathology in patient 1.

### hDAT-K619N shows impaired DA uptake, amphetamine-induced efflux, and surface expression.

**Figure 2:**
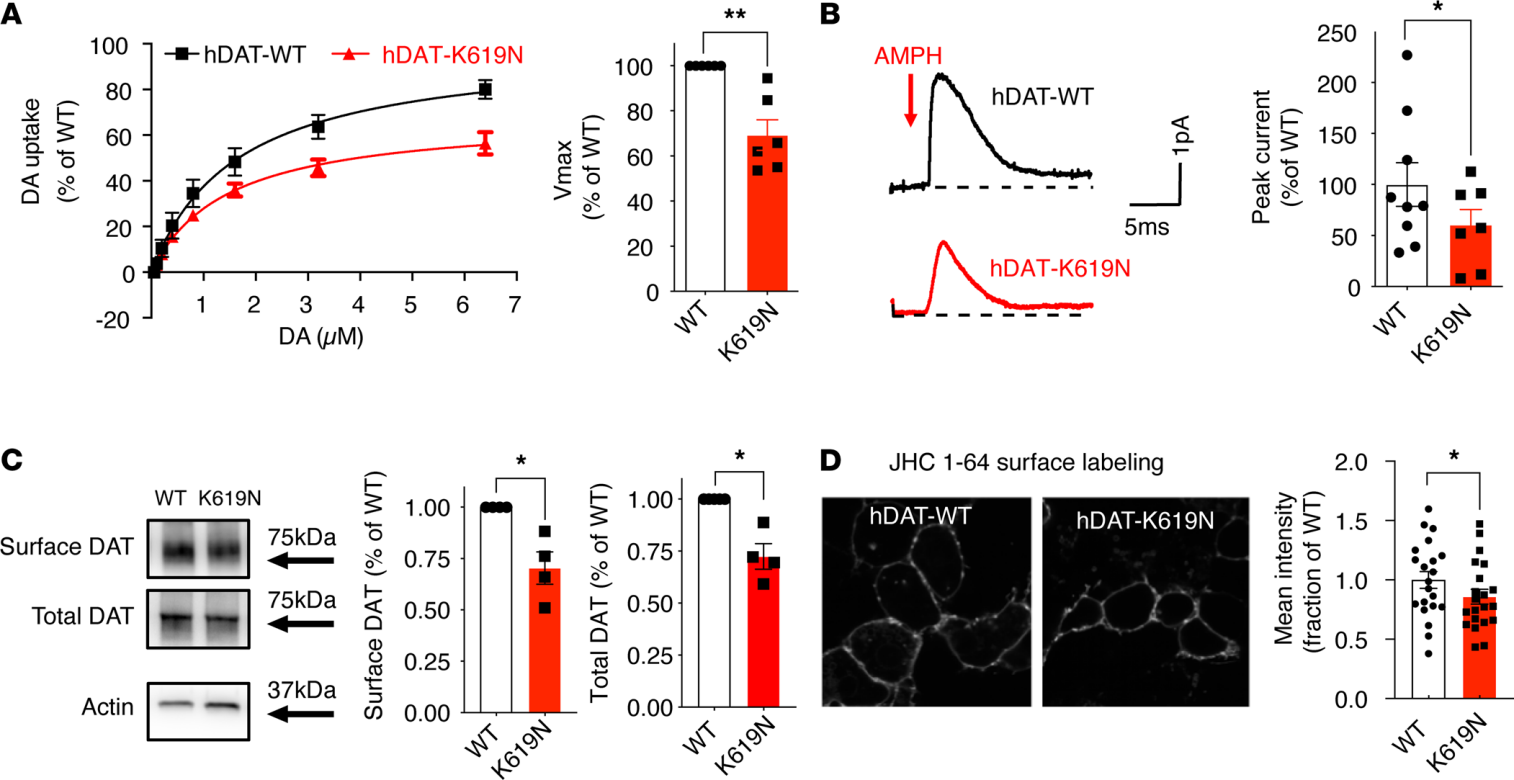
DAT-K619N displays functional impairments and reduced surface expression in vitro. (**A**–**C**) Evaluation of DAT-K619N functions and surface expression in transiently transfected HEK293 cells. (**A**) Functional comparison of DAT-K619N to WT using [^3^H]-dopamine (DA) uptake. Uptake curves (left) are average curves of 6 experiments each performed in triplicate and normalized to the fitted maximal uptake capacity (V_max_) of DAT-WT. The DAT-K619N variant demonstrated reduced V_max_ (right bar diagram) compared with DAT-WT (*P* < 0.01, 1-sample 2-tailed *t* test, *n* = 6) with no accompanying change in *K_m_* (*K_m_* =1.4 ± 0.3 μM for K619N vs. 1.7 ± 0.4 μM for DAT-WT, *P >* 0.05 Student’s *t* test). (**B**) Amphetamine-induced amperometric currents with representative traces of amperometric currents (left) and quantification of the amphetamine-induced peak currents relative to DAT-WT (right). DA-release through DAT-K619N was impaired compared with DAT-WT (*P* < 0.05, 1-sample 2-tailed *t* test, *n* = 9 for WT and *n* = 7 for DAT-K619N). (**C**) Surface biotinylation of transiently transfected HEK293 cells. The amount of DAT-K619N relative to DAT-WT was decreased in the surface and the total protein fractions (*P* < 0.05, 1-sample 2-tailed *t* test, *n* = 4). (**D**) Confocal live imaging of surface-expressed WT and DAT-K619N in HEK239 cells by labeling with the fluorescent cocaine analog, JHC 1-64 (20 nM). Mean intensity of the JCH 1-64 signal was reduced for DAT-K619N relative to DAT-WT. Images were acquired from 4 independent transfections, and intensities were normalized to WT mean intensity for each imaging session (*P* < 0.05, 1-sample 2-tailed *t* test, *n* = 21 images per group). Data are mean ± SEM. *P < 0.05, **P < 0.01.

To investigate the molecular phenotype of hDAT-K619N, we first assessed DA uptake in transiently transfected HEK293 cells. Compared with hDAT-WT, hDAT-K619N demonstrated impaired maximal DA uptake capacity (V_max_ = 69% ± 7% of DAT-WT) without any change in *K_m_* (*K_m_* = 1.4 ± 0.3 μM for hDAT-K619N vs. 1.7 ± 0.4 μM for hDAT-WT, Figure 2A). DAT is known to mediate reverse transport of DA in response to amphetamine, which acts as a competitive substrate and promotes an efflux-prone conformation of DAT (35). Amperometric recordings on transiently transfected HEK293 cells, loaded with DA and stimulated with 10 μM amphetamine, showed reduced amphetamine-induced DA efflux for hDAT-K619N, which generated a peak current of approximately 60% of the hDAT-WT current (Figure 2B).

Next, we addressed whether a reduction in surface expression might be responsible for the functional deficits of hDAT-K619N. Indeed, whole-cell surface biotinylation experiments demonstrated a decrease in surface-expressed hDAT-K619N compared with hDAT-WT (70% ± 8% of hDAT-WT, Figure 2C). The total expression of maturely glycosylated hDAT-K619N was also reduced (79% ± 1% of hDAT-WT, *P* < 0.01), suggesting that the reduced surface level was the result of a reduction in the total cellular level of transporter. The biotinylation data were further supported by assessing surface expression for hDAT-K619N using a fluorescent cocaine analog, JHC 1-64 (20 nM for 20 minutes, room temperature), which allows selective live labeling of the surface-expressed transporters (36, 37). JHC 1-64 produced a clear membrane labeling of hDAT-WT– and hDAT-K619N–expressing cells, but the MFI was reduced in cells expressing hDAT-K619N compared with hDAT-WT (mean JHC 1-64 intensity 86% ± 6% of hDAT-WT), consistent with lower surface levels (Figure 2D).

The functional effects of mutating DAT’s C-terminal PDZ-binding sequence can vary considerably between different expression systems (31). Moreover, we have previously found that disease-associated DAT variants display changes in affinities for ligands and for cotransported Na^+^ and/or Cl^–^ ions and show alterations in Zn^2+^-dependent regulation of DA uptake in COS-7 cells (25). We therefore evaluated DA uptake, [^3^H]-CFT ([^3^H]-2β-carbomethoxy-3β-[4-fluorophenyl] tropane) binding, ligand affinities, and ion coordination and Zn^2+^ regulation in transiently transfected COS-7 cells to further elucidate the molecular phenotype of hDAT-K619N (28). As observed in HEK293 cells, the uptake capacity of hDAT-K619N was reduced by approximately 30%, and we observed a comparable reduction in [^3^H]-CFT binding capacity (B_max_ = 75% ± 5% of hDAT-WT, Supplemental Figure 1). The apparent affinity for neither DA nor [^3^H]-CFT was different between hDAT-WT and hDAT-K619N, and potencies of amphetamine, methylphenidate, and cocaine to inhibit uptake were unaffected for hDAT-K619N. Furthermore, hDAT-K619N showed no changes in coordination of cotransported Na^+^ and Cl^–^ ions, as assessed by DA uptake measurements at increasing Na^+^ and Cl^–^ concentrations, respectively. Likewise, Zn^2+^-dependent regulation of DA uptake was similar for hDAT-WT and hDAT-K619N (Supplemental Figure 1 and Supplemental Table 1). In summary, the functional phenotype of hDAT-K619N could be recapitulated in COS-7 cells, supporting reduced expression and no conformational perturbations of hDAT-K619N.

### Live fluorescence imaging uncovers accelerated turnover rate and increased lysosomal targeting of DAT-K619N.

**Figure 3:**
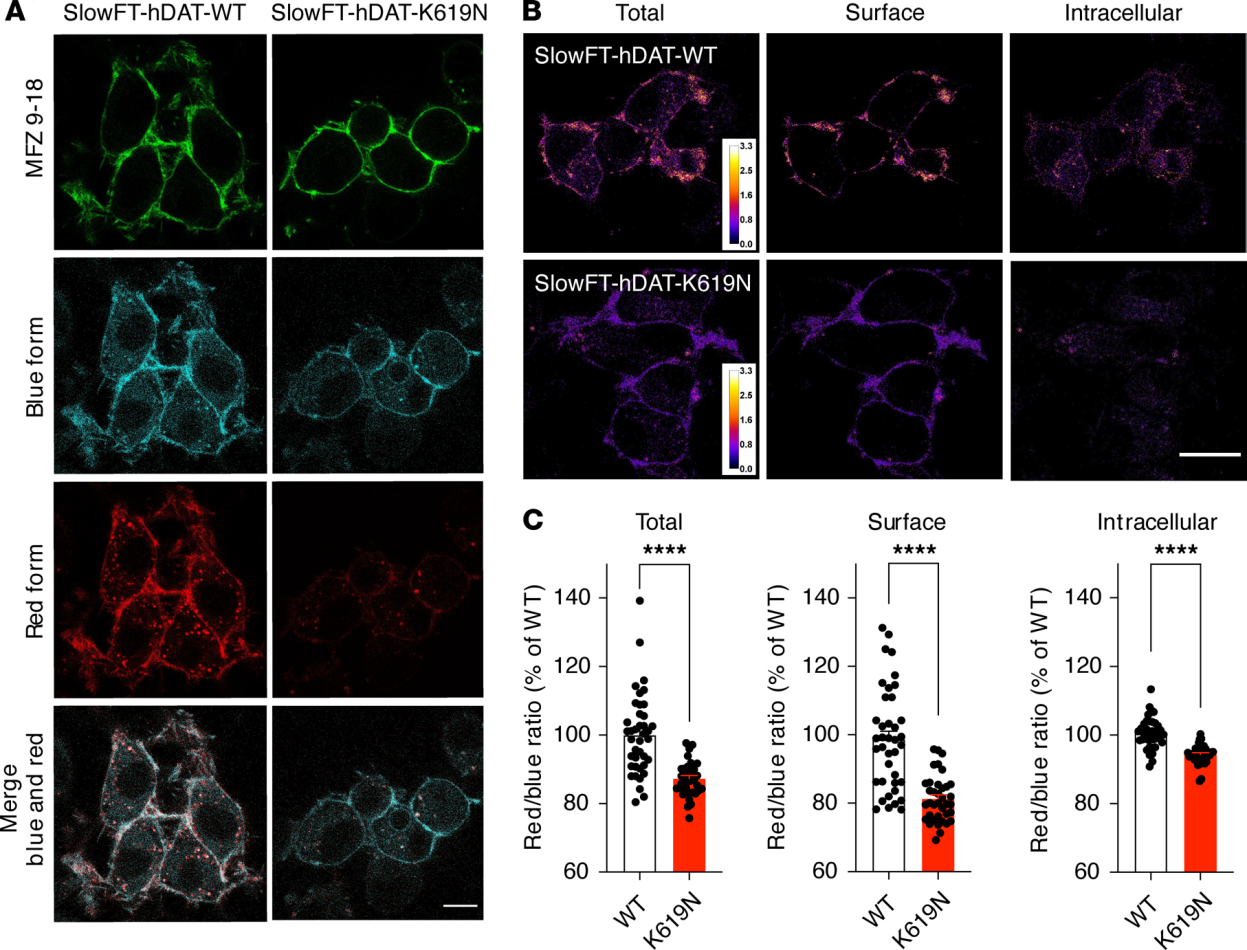
DAT-K619N shows altered cellular processing. Spatiotemporal visualization of DAT-WT and DAT-K619N in transfected CAD cells using N-terminal tagging with the fluorescent timer SlowFT. SlowFT changes color from blue to red over time, reaching maximal blue fluorescence after 9.8 hours and half-maximal red fluorescence after 28 hours (38). Note that the half-life of DAT-WT has been estimated to be approximately 2 days (65). (**A**) Images from live confocal microscopy of SlowFT-DAT-WT and SlowFT-DAT-K619N. A green fluorescent cocaine analog, MFZ 9-18, was used to identify transfected cells and as a surface indicator in postimaging analysis of the surface and intracellular fractions. The blue and red timer forms are shown individually and merged. Note the apparent reduction in the red form of SlowFT-DAT-K619N. (**B**) Pseudocolor images of the red-to-blue ratios, which is a relative measure of age. Warm colors indicate older protein (higher red-to-blue ratios). (**C**) Quantification of mean red-to-blue ratios for SlowFT-DAT-WT and SlowFT-DAT-K619N normalized to the mean red-to-blue ratio of SlowFT-DAT-WT for each imaging session. SlowFT-DAT-K619N was on average younger than SlowFT-DAT-WT, both for the total protein and in the surface and intracellular compartments, consistent with an accelerated turnover of SlowFT-DAT-K619N compared with SlowFT-DAT-WT (*P* < 0.0001, 1-sample 2-tailed *t* test, *n* = 36–39). Data are shown as mean ± SEM. Images are representative of 36 to 39 images from 3 independent experiments. Scale bar: 10 μm. Image analysis was done in ImageJ (NIH; see Methods) ****P < 0.0001.

To obtain further insight into the cellular processing of hDAT-K619N, we took advantage of genetically encoded fluorescent timers (FTs) that change color from blue to red in a time-dependent manner and thereby permit concurrent spatial and temporal visualization of protein trafficking (38). We generated fusion proteins of a blue/red fluorescent timer with reported slow kinetic properties (SlowFT) attached to the N-terminus of hDAT-WT and hDAT-K619N, and we expressed them in a neuronally derived catecholaminergic cell line (Cath.a-differentiated, CAD cells; ref. 39) with relatively large somas, making them suited for live microscopy. Imaging was performed after surface labeling of DAT with an Oregon green–conjugated fluorescent cocaine analog, MFZ 9-18 (36), which enabled identification of transfected cells without direct imaging of the SlowFT (see Methods) and allowed subsequent analysis of surface signal versus intracellularly located DAT. For both SlowFT-hDAT-WT and SlowFT-hDAT-K619N, the fluorescence from the blue and red timer forms colocalized at the membrane but showed only a partial overlap for the intracellular punctate structures (Figure 3A). Interestingly, an inspection of the cells indicated a relatively low intensity of the red form of SlowFT-hDAT-K619N as compared with SlowFT-hDAT-WT. Consistently, quantification of the overall red-to-blue ratio, a relative measure of protein “age,” was reduced for SlowFT-hDAT-K619N compared with SlowFT-hDAT-WT, indicating that hDAT-K619N on average was “younger” than hDAT-WT (Figure 3, B and C). Next, we used the signal of the MFZ 9-18 cocaine analog bound to DAT in the plasma membrane to isolate the surface compartment and thereby analyze the “relative ages” (i.e., red-to-blue ratios) for surface- and intracellularly localized transporters separately. This revealed a reduction in the mean red-to-blue ratio of SlowFT-hDAT-K619N compared with SlowFT-hDAT-WT protein in both compartments (Figure 3C). Of note, we also found that the mean red-to-blue ratio of SlowFT-hDAT-WT in the surface/MFZ 9-18 positive compartment was higher than the red-to-blue ratio of the intracellular compartment, indicating that surface-expressed SlowFT-hDAT-WT, on average, was older than that found intracellularly. By contrast, the mean red-to-blue ratio for SlowFT-hDAT-K619N in the surface compartment was smaller than the mean ratio for the intracellular compartment (Supplemental Figure 2), suggesting that the cellular processing of SlowFT-hDAT-K619N differed from SlowFT-hDAT-WT. The overall reduction in mean age of SlowFT-hDAT-K619N compared with SlowFT-hDAT-WT, as well as the increase in mean age of SlowFT-hDAT-K619N residing in intracellular compartments relative to that on the surface, was consistent with the K619N mutation leading to an accelerated turnover rate. We further rationalized that an accelerated turnover of hDAT-K619N likely would be associated with enhanced lysosomal degradation. Accordingly, we expressed mCherry-tagged versions of hDAT-WT and hDAT-K619N in CAD cells and concurrently visualized the lysosomes with LysoTracker. Quantification of the mCherry mean intensity confirmed a reduction for mCherry-hDAT-K619N compared with mCherry-hDAT-WT (Supplemental Figure 3). Using Manders coefficients to quantify the fraction of the total mCherry signal that overlapped with lysosomes, we found that the fractional overlap between mCherry-hDAT-K619N and LysoTracker-positive compartments was significantly larger (~33%) than for mCherry-hDAT-WT (fractional overlap = 0.060 ± 0.006 for hDAT-WT vs. 0.080 ± 0.007 for hDAT-K619N, Supplemental Figure 3), indicating the hDAT-K619N variant may be subject to enhanced lysosomal degradation.

### Expression of hDAT-K619N in Drosophila causes age-dependent changes in locomotor responses.

**Figure 4:**
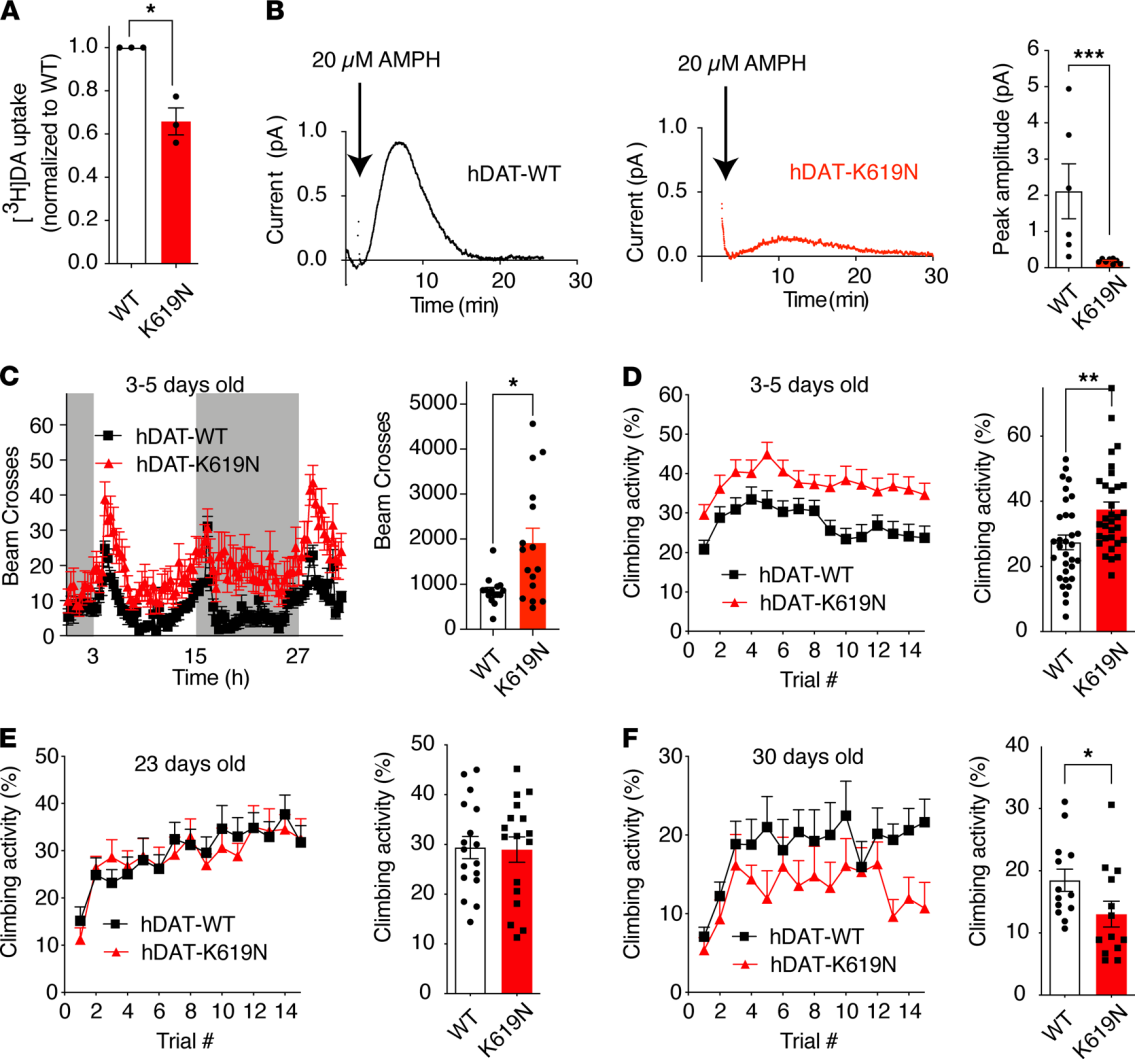
Expression of DAT-K619N drives dopaminergic dysfunction and progressive locomotor disturbances in *Drosophila*. (**A**) Amperometric recordings of amphetamine-induced DA efflux in whole brains from *Drosophila* expressing WT human DAT (hDAT) or hDAT-K619N. Representative traces and quantification of peak amperometric currents are shown. DAT-K619N flies displayed markedly reduced amphetamine-induced efflux (*P* < 0.001, Mann-Whitney test, *n* = 6–8). (**B**) DA uptake (200 nM for 15 minutes) into isolated whole fly brains normalized to DAT-WT in each experiment. DA uptake was compromised in brains from the DAT-K619N strain compared with DAT-WT (*P* < 0.05, 1-sample 2-tailed *t* test, *n* = 3). (**C**) Locomotor activity recordings of 3- to 5-day-old flies over 30 hours showed that DAT-K619N flies were hyperactive during both light phases (light bars) and dark phases (dark bars), with a 125% increase in mean number of beam breaks (849 ± 82 for DAT-WT vs. 1910 ± 320 for DAT-K619N, *P* < 0.05, Mann-Whitney test, *n* = 15–16 flies). (**D**–**F**) Assessment of negative geotactic crawling response in flies that were 3 to 5 days old (**D**), 23 days old (**E**), and 30 days old (**F**). The curves show climbing activity over 15 consecutive trials. Bar diagrams compare the mean climbing activity of the 15 trials; 3- to 5-day-old DAT-K619N flies displayed a hyperactive phenotype (*P* < 0.01, Mann-Whitney test, *n* = 32 cohorts), which was absent at day 23 (*P >* 0.05, Mann-Whitney test, *n* = 17 cohorts) and at day 30, the DAT-K619N flies showed reduced climbing activity compared with DAT-WT–expressing flies (*P* < 0.05, Mann-Whitney test, *n* = 13 cohorts). This indicates that the DA dysfunction in DAT-K619N flies progressed over time. *P < 0.05; **P < 0.01; ***P < 0.001.

Dopaminergic control of locomotion and exploration is highly conserved across species, including *Drosophila*, and drosophila-based models have been shown to capture core features of Parkinson disease and neuropsychiatric disorders (19, 23, 40, 41). To address whether hDAT-K619N causes functional and behavioral alterations in vivo, we used the Gal4/UAS system to generate knockin (KI) lines, expressing a single copy of either EGFP-tagged or untagged hDAT-WT or hDAT-K619N in dopaminergic neurons of *Drosophila* DAT (dDAT) KO flies. Confocal imaging of EGFP-tagged hDAT-WT and hDAT-K619N showed comparable expression of both transgenes in the somatic regions (PPL1) and in the fan-shaped body, which is a major projection target of these neurons (Supplemental Figure 5). Measurement of DA uptake in intact isolated brains from the flies expressing untagged transporters, however, showed that uptake into hDAT-K619N brains was reduced to 65.8% ± 6% of hDAT-WT brains (mean uptake hDAT-WT: 333 ± 5 fmol/brain vs. 219 ± 20 fmol/brain in DAT-K619N, Figure 4A). Moreover, in the hDAT-K619N-KI flies, amphetamine-induced DA efflux was reduced by approximately 80% (DA peak current) compared with hDAT-WT as determined by amperometric recordings (Figure 4B). Importantly, the impairments in DAT function imposed by the K619N mutation were associated with changes in locomotor activity. Thus, when the flies were placed in activity chambers, more beam breaks were recorded for hDAT-K619N flies compared with hDAT-WT flies during the light and dark cycles (Figure 4C). In addition, a test of startle-induced negative geotactic response demonstrated enhanced climbing activity for hDAT-K619N flies (Figure 4D). To monitor whether the enhanced climbing response persisted as the flies aged, we also evaluated the climbing response in 23- and 30-day-old flies. Remarkably, we found that the hyperactive climbing response of hDAT-K619N–expressing flies was lost in 23-day-old flies, and by day 30 the hDAT-K619N flies were showing deficient climbing compared with hDAT-WT–expressing flies. This finding suggests that the behavioral consequences of hDAT-K619N may be age dependent or progressive in nature (Figure 4, D–F).

### Unilateral nigral overexpression of hDAT-WT but not hDAT-K619N generates rotational bias in response to amphetamine.

**Figure 5:**
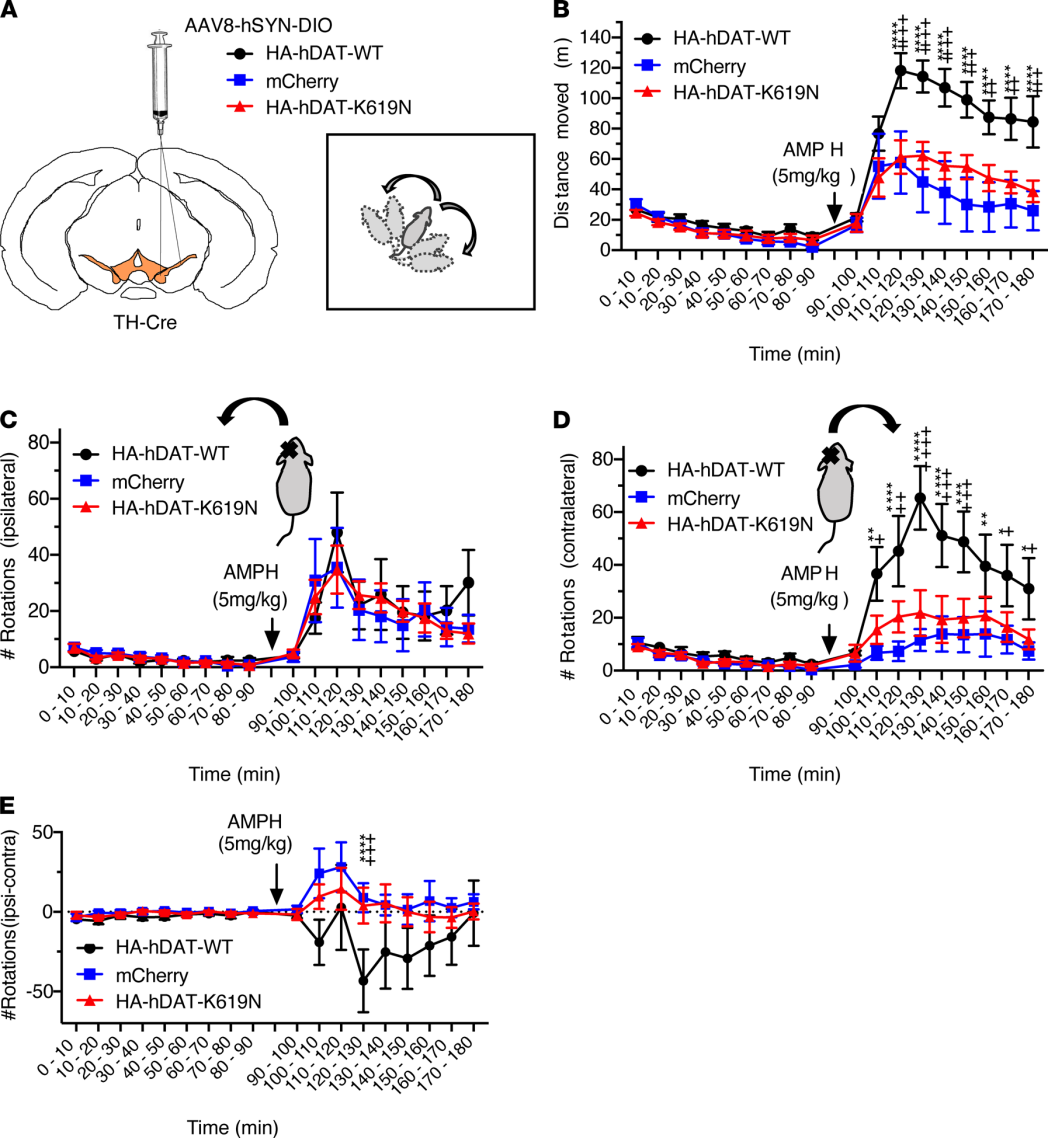
Amphetamine-induced rotations reveal differential DA-controlled behaviors following unilateral expression of DAT-WT and DAT-K619N. The effect of HA-DAT-WT and HA-DAT-K619N on striatal DA homeostasis was compared by unilateral expression and open-field assessment of amphetamine-induced rotations. (**A**) TH-Cre mice were injected unilaterally in substantia nigra with AAV encoding either HA-DAT-WT, HA-DAT-K619N, or mCherry as a control plasmid. Amphetamine-induced rotations were evaluated in the open-field setup 3 weeks after injections. (**B**) Distance travelled in open-field chambers 90 minutes before and after amphetamine injections (5 mg/kg, i.p.). Mice injected with HA-DAT-WT differed from HA-DAT-K619N–injected mice by driving enhanced activity relative to both mCherry-injected and HA-DAT-K619N–injected mice (repeated measures 2-way ANOVA followed by Holm-Šídák multiple-comparison test, *n* = 7–9 mice). (**C**) Evaluation of ipsilateral rotations showed no difference between mice injected with HA-DAT-WT, HA-DAT-K619N, or mCherry upon amphetamine exposure (*P >* 0.05, repeated measures 2-way ANOVA). (**D**) The number of contralateral rotations after amphetamine treatment was enhanced only in mice expressing HA-DAT-WT and not in HA-DAT-K619N–expressing mice, supporting the differential effects of HA-DAT-K619N and HA-DAT-WT in vivo. (**E**) Rotational laterality assessed as ipsilateral-contralateral rotations before and after amphetamine injections. Only HA-DAT-WT–injected mice displayed lateralized rotational behavior (2-way ANOVA followed by Holm-Šídák multiple-comparison test, *n* = 7–9 mice). In panels **B**, **D**, and **E**, plus signs mark statistical differences between HA-DAT-WT and HA-DAT-K619N, and asterisks mark statistical differences between HA-DAT-WT and mCherry. No differences were found between HA-DAT-K619N and mCherry. ****/^++++^*P* < 0.0001, ***/^+++^*P* < 0.001, **/^++^*P* < 0.01, */^+^*P* < 0.05). All data are shown as mean ± SEM.

Overexpression of WT DAT in mice has been shown to produce enhanced locomotor responses specifically to amphetamine, whereas heterozygote DAT-KO mice with 50% DAT expression have preserved amphetamine-induced locomotion (42, 43). To directly compare the effect of virally expressing hDAT-K619N or hDAT-WT on striatal DA function in WT mice, we unilaterally expressed hDAT-K619N or hDAT-WT in midbrain dopaminergic neurons and determined how these transgenes affected amphetamine responses (5 mg/kg) in the open-field test (44, 45). To enable specific detection of the virally encoded transgenes, an HA-tag was fused to the N-terminal of hDAT-WT (HA-hDAT-WT) and hDAT-K619N (HA-hDAT-K619N). Adeno-associated virus (AAV) with a double-floxed inverse open reading frame (DIO) was stereotactically injected unilaterally in substantia nigra of tyrosine hydroxylase–Cre (TH-Cre) mice to obtain Cre-dependent expression of either HA-hDAT-WT or HA-hDAT-K619N selectively in TH-expressing midbrain neurons. As an additional control, we injected mice with an AAV construct encoding Cre-dependent mCherry to account for unspecific effects related to surgery and protein overexpression. Immunohistochemical stainings of midbrain and striatal slices from TH-Cre mice injected with AAV8-DIO-HA-hDAT-WT, AAV8-DIO-HA-hDAT-K619N, or AAV8-DIO-mCherry confirmed expression of the viral transgenes in TH-positive neurons and revealed the presence of both HA-hDAT-WT and HA-hDAT-K619N in TH-labeled projections/axonal terminals in the striatum (Supplemental Figure 4). Interestingly, we observed that the effect of overexpressing HA-hDAT-K619N differed markedly from HA-hDAT-WT overexpression. Thus, mice overexpressing HA-hDAT-WT but not HA-hDAT-K619N displayed larger amphetamine-induced hyperlocomotion than control mice injected with AAV encoding mCherry (Figure 5, A and B). Moreover, the unilateral overexpression of HA-hDAT-WT established a rotational laterality with more contralateral rotations upon amphetamine stimulation, whereas overexpression of HA-hDAT-K619N or mCherry did not establish amphetamine-induced rotational laterality (Figure 5, C–E). Note that in lesion studies, amphetamine induces rotations toward the lesioned side (44). Collectively, these findings indicate that the K619N mutation causes functional changes to DAT function that can translate into altered control of extracellular dopamine.

### hDAT-K619N exerts dominant-negative impairments on DA uptake in vitro and in vivo.

**Figure 6:**
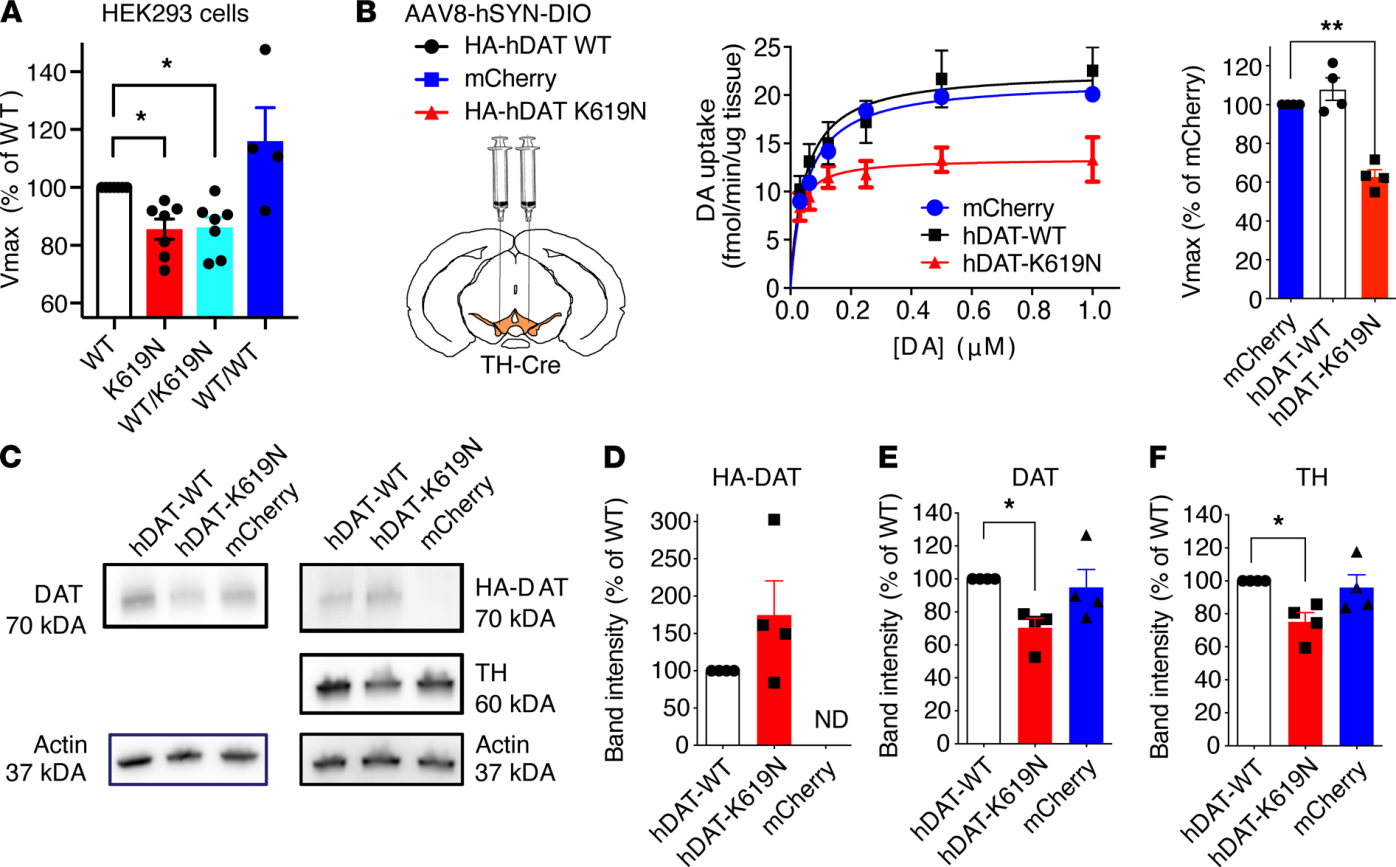
DAT-K619N exerts a dominant-negative effect on DAT-WT. (**A**) Evaluation of dominant-negative effects of DAT-K619N in vitro. DA uptake was measured in HEK293 cells, cotransfected with equal amounts (1.5 μg) of DAT-K619N and DAT-WT, and compared with cells transfected only with DAT-WT (1.5 μg + 1.5 μg empty vector), DAT-K619N (1.5 μg + 1.5 μg empty vector), or with 3 μg of DAT-WT as a control. Each experiment was performed in triplicate and normalized to V_max_ of DAT-WT (1.5 μg + 1.5 μg empty vector). The V_max_ of DAT-K619N/DAT-WT cotransfected cells (1.5 μg + 1.5 μg) was reduced relative to DAT-WT (1.5 μg + 1.5 μg empty vector) to a level similar to DAT-K619N alone (*P < 0.016, 1-sample 2-tailed *t* test, Bonferroni-adjusted significance level [= 0.05/3] *n* = 4–7) indicating dominant-negative actions of hDAT-K619N. (**B**) Evaluation of dominant-negative effects in vivo performed by overexpressing HA-hDAT-WT or HA-hDAT-K619N selectively in dopaminergic neurons, using bilateral midbrain AAV injections in TH-Cre mice, and performing [^3^H]-DA uptake on striatal synaptosomes. Injections with AAV encoding mCherry was used for comparison with endogenous DA uptake levels. Uptake curves are average curves of 4 experiments each performed in triplicate. Quantification of V_max_ normalized to mCherry showed HA-hDAT-K619N but not HA-hDAT-WT reduced DA uptake below endogenous uptake capacity (*P < 0.005, 1-sample 2-tailed *t* test, Bonferroni-adjusted significance level: **P < 0.01/2; *n* = 4. (**C**) Western blot analysis of striatal synaptosomal preparations. Representative blots for total DAT, HA-DAT, and TH are shown. Specific detection of HA-hDAT-WT and HA-hDAT-K619N using anti-HA antibody confirmed both constructs were trafficked to striatal terminals. (**D**–**F**) Quantification of relative expression levels revealed a reduction in both DAT (**E**) and TH (**F**) in HA-hDAT-K619N–injected mice (*P < 0.025, 1-sample 2-tailed *t* test, Bonferroni-adjusted significance level [= 0.05/2], *n* = 4); HA-DAT levels did not differ significantly between HA-DAT-WT and HA-DAT-K619N–injected mice (*P* = 0.21, 1-sample 2-tailed *t* test, *n* = 4).

Given the heterozygote carrier status of the identified patients, an important question is whether a single hDAT-K619N allele is sufficient to drive DA dysfunction and thereby potentially confer disease risk. DAT has been shown to form oligomers (46), which could allow hDAT-K619N to directly influence WT-hDAT function or trafficking in heterozygote carriers. We therefore cotransfected HEK293 cells with equal amounts of DNA (1.5 μg) of both hDAT-K619N and hDAT-WT and compared the resulting DA uptake capacity with that obtained when cells were transfected with only hDAT-WT or hDAT-K619N, using an empty vector to keep the total amount of DNA constant (3 μg). Notably, the uptake capacity of cotransfected cells (1.5 μg hDAT-WT plus 1.5 μg hDAT-K619N) was significantly reduced compared with hDAT-WT single transfection (1.5 μg hDAT-WT plus 1.5 μg empty vector) and similar to that of cells transfected only with hDAT-K619N (V_max_ = 86% ± 3% of hDAT-WT for WT/KN cotransfection and 87% ± 3% of hDAT-WT for hDAT-K619N alone). As a control, we transfected cells with 3 μg of only hDAT-WT, which did not establish a similar reduction in uptake (V_max_ = 116% ± 9% of 1.5 μg hDAT-WT), suggesting that the hDAT-K619N variant has a dominant-negative effect on hDAT-WT function (Figure 6A).

We next sought to evaluate the dominant-negative effect of the hDAT-K619N variant in vivo. To do this, we expressed HA-hDAT-K619N in dopaminergic neurons of WT mice, i.e., on top of the endogenously expressed DAT, thereby mimicking the heterozygous genotype of the patients. TH-Cre mice received bilateral injections of AAVs encoding either HA-hDAT-K619N, HA-hDAT-WT, or mCherry in the ventral tegmental area, and striatal synaptosomes were prepared to compare the resulting ^3^H-DA uptake capacity. Strikingly, mice expressing HA-hDAT-K619N showed a pronounced reduction (~40%) in DA uptake relative to the endogenous uptake capacity derived from mice expressing the mCherry reporter, indicating a dominant-negative effect of hDAT-K619N in vivo. By contrast, mice injected with HA-hDAT-WT did not show significantly altered DA uptake relative to mice injected with mCherry (Figure 6B). The reduction in V_max_ observed in mice expressing HA-hDAT-K619N was not accompanied by significant changes in *K_m_* (*Km*_ mCherry_ = 55 ± 9 nM, *Km*_ K619N_ = 24 ± 6 nM, and *Km*_ WT_ = 57 ± 18 nM, *P* > 0.05, 1-way ANOVA, with Holm-Šídák posttest, *n* = 4). Using the HA-tag, Western blot analysis of the synaptosomal fractions supported the presence of both HA-hDAT-WT and HA-hDAT-K619N in the striatum (Figure 6, C and D). Interestingly, quantification of the total DAT signal revealed an approximately 30% reduction in mice injected with HA-hDAT-K619N compared with mice injected with HA-hDAT-WT (Figure 6, C and E). Moreover, we observed a reduction in TH expression in the synaptosomal fractions from HA-hDAT-K619N–expressing mice compared with HA-hDAT-WT (75% ± 6% of HA-DAT-WT, Figure 6, C and F), further supporting that the HA-hDAT-K619N variant generated changes in the DA system that were distinct from HA-hDAT-WT. Together, the viral overexpression of hDAT-K619N suggests that the K619N mutation alters DA uptake dynamics in vivo via dominant-negative impairments of DAT function.

### Population genetics of the DAT-K619N variant.

With the increasing use of gene or whole-exome sequencing in clinical practice, a critical challenge will be to provide accurate genetic counseling upon identification of rare or novel variants. Functional data are of utmost importance for genetic counseling but do not provide estimates of effect sizes. Like previously identified DAT coding variants (25), hDAT-K619N (rs200712598) can be found in genetic databases of the general population, with a reported overall carrier frequency among northern Europeans of approximately 0.0012 in the Genome Aggregation Database (29). Unfortunately, these databases do not hold information about disease diagnoses for the carriers. To take initial steps to derive effect size estimates for the hDAT-K619N variant in psychiatric disease, we looked up the occurrence of hDAT-K619N in 19,851 exome-sequenced samples from the iPSYCH consortium case-cohort covering 5 major mental disorders: ADHD, ASD, schizophrenia, depression, and bipolar disorder (47). A total of 17,339 samples were included in the analysis, comprising 4885 controls and 12,327 cases diagnosed with at least 1 of the following diseases: ADHD (*n* = 4548), ASD (*n* = 4937), schizophrenia (*n* = 3052), single-episode or recurrent depression (*n* = 2623), or bipolar disorder (*n* = 1313; Table 2). The hDAT-K619N variant was observed in 5 controls and 21 cases, corresponding to carrier frequencies of 0.0010 and 0.0017. The median and mean age of DAT-K619N carriers among cases and controls were comparable (case-carriers: mean age = 25.68 years, median age = 25.42 years, SEM = 0.98; control-carriers: mean age = 28.75 years, median age = 27.36 years, SEM = 1.73. A carrier-based association analysis did not establish a disease association across all diagnostic groups (*P* = 0.29, OR: 1.7, 95% CI: 0.68–4.3). Analysis of individual diagnostic groups, however, showed a general trend of increased ORs across all diagnostic categories and demonstrated a nominally significant association with bipolar disorder (Table 2). It is important to note that the iPSYCH sample includes relatively young individuals (47) (born between 1981 and 2005). Because new diagnosis and/or rediagnosis are likely to occur in the future, the disease frequencies might be underestimated.

## Discussion

Diseases involving DA dysfunction span neurological disorders, such as parkinsonism, and widespread neuropsychiatric diseases, such as ADHD, ASD, bipolar disorder, schizophrenia, and depression (1, 3–6). DAT is a powerful regulator of DA neurotransmission, but the genetic and mechanistic link between insult to DAT function and DA-related pathologies is not clear. Specifically, the role of heterogeneity for rare coding variants in parkinsonism and/or neuropsychiatric disease remains elusive. Here, we have provided new insight into the link between DAT dysfunction and human disease by employing a translational approach to investigate the rare coding variant hDAT-K619N, which we identified in an index patient with early-onset parkinsonism as well as in multiple patients with psychiatric disorders. We combined detailed clinical data, neuroimaging, and large-scale exome data to address the occurrence and phenotypic spectrum of hDAT-K619N carriers and employed in vitro studies alongside in vivo and ex vivo investigations in mouse and *Drosophila* models of hDAT-K619N to uncover the functional consequences of the rare but recurring coding variant with apparent dominant-negative properties.

Our in vitro studies in heterologous cells revealed a parallel reduction in DA uptake, amphetamine-induced DA efflux, and [^3^H]-CFT–binding capacity for hDAT-K619N compared with hDAT-WT, which, together with preserved Zn^2+^-dependent regulation and normal ligand and ion affinities, suggested that the K619N mutation changes cellular DAT processing without imposing conformational alterations that impair catalytic activity. The phenotype was supported by whole-cell surface biotinylation experiments and surface staining of DAT in live cells, which demonstrated a decrease in surface-expressed hDAT-K619N compared with hDAT-WT. Our live-cell imaging of hDAT-K619N coupled to a fluorescent timer showed that the hDAT-K619N protein, on average, was younger (smaller red-to-blue ratio) than hDAT-WT. We also observed increased localization of mCherry-tagged hDAT-K619N to lysosomes in live CAD cells, suggesting that hDAT-K619N is subject to accelerated turnover leading to lower expression of active transporter. Interestingly, the DAT C-terminus, including the C-terminal PDZ-binding motif that K619 is part of (L618, K619, and V620), has been subject to several previous mutational studies (30–32, 48, 49). We have previously alanine-substituted DAT’s PDZ-binding sequence (DAT-AAA) to disrupt interactions with yet unknown PDZ domain scaffold proteins. This led to reduced expression and enhanced degradation of DAT-AAA, and, hence, a molecular phenotype similar to what we found for hDAT-K619N (31). Other mutations have implicated the same PDZ-binding sequence in folding and ER export (30, 32), collectively suggesting that the DAT C-terminus is highly sensitive to mutational changes and that an intact C-terminus is critical for biosynthesis, trafficking, and turnover of the DAT protein.

Our subsequent in vivo and ex vivo experiments indicated that the hDAT-K619N variant can give rise to changes in DA function of potential pathological importance. The experiments also affirmed that the molecular phenotype is markedly more pronounced in vivo, consistent with a previous finding for the DAT-AAA mutation (31). Thus, we found that whole-brain DA uptake was reduced by approximately 35% and amphetamine-induced DA efflux was reduced by more than 80% in flies expressing the hDAT-K619N variant compared with hDAT-WT. Moreover, young flies expressing hDAT-K619N demonstrated hyperlocomotion and an enhanced negative geotactic response compared with hDAT-WT–expressing flies. In mice, the differential effect of hDAT-K619N and hDAT-WT was sufficient to establish differences in DA-directed behaviors. Rotational behavior is a sensitive measure of asymmetry in striatal DA (44, 45), and we observed that although unilateral HA-hDAT-WT expression led to increased overall locomotor activity and contralateral rotational bias in response to amphetamine, this was not observed in mice expressing HA-hDAT-K619N or the mCherry control.

Another important finding was the approximately 40% reduction in striatal synaptosomal uptake seen upon bilateral viral expression of HA-hDAT-K619N but not upon expression of HA-hDAT-WT or mCherry in midbrain DA neurons. This suggests that hDAT-K619N is capable of inhibiting DA uptake in a dominant-negative manner, as was observed in vitro, too (Figure 5). From a mechanistic perspective, the dominant-negative effect of hDAT-K619N is likely linked to transporter oligomerization (49), meaning that accelerated degradation of hDAT-K619N through interaction with hDAT-WT might lead to concomitant accelerated degradation of hDAT-WT (49). Indeed, a previous study found that nonfunctional C-terminal truncations in DAT can reduce surface expression of the DAT-WT in a dominant-negative fashion, and the authors concluded that this was the result of oligomeric interactions between the mutants and the WT protein (49). It should also be noted that although we confirmed the presence of both HA-hDAT-K619N and HA-hDAT-WT in striatum and directly compared their impact on DA uptake, we did not derive an isolated measurement of the HA-hDAT-K619N and HA-hDAT-WT functionality in the mice. The DAT-KO mice background would be more ideal for such functional investigations, as well as for studies of differential trafficking.

The dominant-negative action of hDAT-K619N is particularly noteworthy because patient 1, to our knowledge, is the first example of a heterozygote carrier of a coding DAT variant with early-onset parkinsonism. To date, all previously described patients with DTDS have been either homozygous or compound heterozygous for disruptive variants in *SLC6A3* (13, 15–17, 50, 51). Thus, the current data might conceptually and mechanistically expand the allelic disease spectrum of DTDS from biallelic nonsynonymous variants to single susceptibility/disease alleles. It is not clear exactly how much DAT activity is required to maintain human health. In classical DTDS, where biallelic mutations cause complete loss of DAT function, the disease manifests in early infancy (14). Adding to this information, we recently described an atypical case of DTDS where the patient was compound heterozygous for partially disruptive DAT variants with an estimated residual DAT activity of approximately 30% (17). This patient, like patient 1 in the present study, had early-onset parkinsonism and neuropsychiatric disease and was classified as having atypical DTDS. Also, 3 brothers with atypical juvenile-onset DTDS have been described for which the predicted DAT activity was approximately 8% (14, 16), strongly supporting a phenotypic spectrum determined by residual uptake activity of DAT. The final level of DAT uptake capacity in human heterozygote carriers of hDAT-K619N is difficult to predict and may vary between people and be influenced by unknown gene-gene or gene-environment interactions. Our functional studies strongly suggest that heterozygosity for hDAT-K619N would considerably compromise, although not completely abolish, DA uptake, as further supported by the DAT-SPECT scans of our index patient. The residual DA uptake could be important not only for the delayed onset of patients 2’s motor symptoms, compared with classical cases of DTDS, but also the positive treatment effects of l-DOPA.

Another question is whether impairments in DAT function can contribute to a neurodegenerative process. The SPECT scans performed 7 years apart showed accelerated loss of DAT binding compared with age-matched controls, indicative of progressive dopaminergic neurodegeneration. This finding aligns with previous data from our other patient with atypical adult-onset DTDS, in whom we also found evidence for dopaminergic degeneration (17). Interestingly, when HA-hDAT-K619N was expressed in WT mice, we observed not only a decrease in uptake, but also a parallel reduction in DAT and TH levels. Reduction in striatal TH is often considered indicative of dopaminergic denervation or neurodegeneration, which is further strengthened by the parallel reduction in total DAT (52–55). Another not mutually exclusive explanation for the reduced TH level in HA-hDAT-K619N–expressing mice is a D2-mediated downregulation of TH activity occurring secondary to the impairments in DAT function and elevated extracellular DA levels (56). We should also note that when expressing HA-DAT-K619N unilaterally in nigrostriatal neurons, we did not observe amphetamine-induced ipsilateral rotational bias, an often-used measure of motor impairments and functional recovery following unilateral lesions. At the same time, it is important to point out that this phenotype is an indirect correlate for dopaminergic neurodegeneration, and it only manifests when approximately 50% or more of nigral DA neurons are lost (44). Of further interest, the behavioral data on flies expressing hDAT-K619N suggest that the dopaminergic dysfunction caused by this mutant is not static but rather progressive in nature. In the hDAT-K619N flies, we observed an initial hyperactive climbing response, which disappeared over time, and at day 30, the climbing activity had even dropped below that of WT hDAT-KI flies (Figure 4). Further studies are needed to elucidate whether the loss of DA neurons that have now been observed in 2 independent cases of atypical DTDS reflects a shared pathophysiological mechanism. Our findings provide additional support to the notion that DAT dysfunction may be directly involved either in initiating neurodegenerative processes in DA neurons or in aggravating the selective vulnerability of these highly specialized neurons.

The genetic component of neuropsychiatric diseases has been firmly established (57) and shown to involve common variants and rare variants (57–59). Identification of causal variants and interrogation of their functional impact are critical next steps for advancing our disease understanding. *SLC6A3* has not been identified as a risk gene in GWAS studies of psychiatric disease or neurological disorders (12). This, however, does not infer that DAT dysfunction cannot generate disease-causing, disease-contributing, or disease-modulating processes. Rather, it reflects the absence of common risk variants. Several rare coding DAT variants with functional deficits in vitro and in vivo have been identified in patients with psychiatric disorders (17–27), but the causal link and effect size of putative risk alleles is still elusive because the family trees and cohort sizes have been too small for even approaching meaningful linkage or association analysis. With the increasing use of exome sequencing in clinical practice, the number of novel or rare coding variants in susceptibility genes will continue to increase. It is therefore of utmost importance to obtain the most accurate data for interpretation and counseling, even if set data do not provide simple answers. We have, to the best of our knowledge, carried out the first association analysis of a coding DAT variant using large-scale exome data. This analysis found that the ORs for the hDAT-K619N allele were increased across all the diagnostic categories and identified a nominally significant association with bipolar disease with an estimated OR of 3.7 (95% CI: 1.2–11.3). For comparison, rare copy number variants, affecting multiple genes, have a wide range of reported ORs from 2 to 57 in patients with schizophrenia (60). The identification of hDAT-K619N carriers among healthy individuals and patients with different neuropsychiatric diseases is not surprising, given that pleiotropic effects and incomplete penetrance have been repeatedly described for common and rare variants in psychiatric disorders. It therefore seems likely that the phenotypic outcome of hDAT-K619N is dependent on other factors, such as genetic background and environmental factors (59). Nonetheless, larger samples are needed to establish true associations and derive more precise effect size estimates. It should also be mentioned that our study population is relatively young, and it would be relevant to have follow-up studies to uncover rediagnoses, potential new incidences of disease among control participants, and investigations of movement disorders later in life.

In summary, our investigations identified a rare coding DAT variant in the critical C-terminal PDZ-binding motif that when present on a single allele can cause dopaminergic dysfunction through a dominant-negative effect, thereby conceivably conferring risk for neuropsychiatric disease and neurodegenerative early-onset parkinsonism. This insight should be crucial in future efforts aimed at further understanding how altered DAT function contributes to DA pathologies and how we can develop better treatment strategies for these diseases.

## Methods

### Patients’ clinical assessment and procedures.

Patient 1 was identified in a previously described referral-based hospital cohort of patients with early-onset parkinsonism or related atypical movement disorders (17). Patient 2 was part of the Simons Simplex Collection, which consists of sequencing data from more than 2000 families with ASD (33). Medical case notes on patient 1 were reviewed to delineate the clinical history and prior treatments. Video recordings were made for documentation of clinical features. A DAT-SPECT scan was made for comparison with a DAT-SPECT scan made 7 years prior. The 2 scans were performed on the same scanner and meticulously in the same manner. The patient received 200 mg sodium perchlorate i.v. to block uptake of free ^123^I in the thyroid gland before i.v. injection of 185 MBq [^123^I]-2β-carbometoxy-3β-[4-iodophenyl]-*N*-[3-fluoropropyl]nortropane ([^123^I]FP-CIT; GE Healthcare). The SPECT data acquisition was begun after exactly 3 hours with a PRISM 3000XP (Marconi, Phillips) triple-headed gamma camera equipped with low-energy, ultra-high-resolution fan-beam collimators. Image reconstruction was performed using iterative reconstruction with scatter and nonuniform attenuation correction. Pixel size after reconstruction was *x*, *y* = 3.1 × 3.1 mm^2^ with a slice thickness of 6.2 mm. From the individual reformatted data set, the 2 neighboring striatal slices with maximum counts per pixel were summed and used for image analysis. DAT availability was quantified as the ratio between specifically bound radioligand and nondisplaceable radioligand for 5 regions of interest representing striatum (caudate nucleus and putamen) bilaterally and in the occipital cortex for calculation of nonspecific binding. The following uptake ratios were calculated: a) specific uptake in striatum = (striatum – occipital cortex)/occipital cortex, b) specific uptake in putamen = (putamen – occipital cortex)/occipital cortex, and c) putamen/caudate nucleus to evaluate symmetry/asymmetry of DAT availability.

### iPSYCH exome-sequencing data.

The exome-sequencing data used in the present study are from the iPSYCH consortium’s first-phase genotyping of a nationwide Danish birth cohort of 1,472,762 individuals born between May 1, 1981, and December 31, 2005. The iPSYCH case-cohort design has been described in greater detail previously (47). The study was approved by the Danish Data Protection Agency. Procedures for exome sequencing and sample and variant quality control are described in Supplemental Methods. After quality control, hDAT-K619N carrier status was available for 17,339 samples.

For carrier-based association analysis, individuals within the case and control samples were categorized as either carriers or noncarriers, and 2 × 2 contingency tables with 2-sided Fisher’s exact tests were applied to test for carrier-based disease associations. A total of 6 association tests were performed, and study-wide significance was accordingly adjusted to *P* < 0.0083.

### Cell culturing and transfection.

Human embryonic kidney cells, CAD cells, and COS-7 cells were maintained and transfected as previously described (17, 25). Assays were conducted 36 to 48 hours after transfection. All in vitro experiments, except live cell imaging of fluorescently tagged constructs, were carried out on heterologous cells transiently transfected with pRC/CMV-hDAT-WT or hDAT-K619N. Fluorescently tagged constructs (mCherry and slowFT) were generated by N-terminal fusion of the fluorescent protein to a synthetic hDAT in pcDNA3. The slow fluorescent timer was provided by Vladislav Verkhusha (Albert Einstein College of Medicine, Bronx, New York, USA). The K619N mutation was introduced in the various hDAT background constructs using either QuickChange site-directed mutagenesis (Stratagene) or 2-step PCR. All constructs were verified by sequencing.

### Saturation [^3^H]-DA uptake.

[^3^H]-DA uptake experiments for determination of kinetic constants and evaluations of dominant-negative actions of hDAT-K619N were carried out as previously described (25), using a 10 × 2-fold dilution row containing a mix of [2,5,6]-(^3^H)-DA (PerkinElmer Life Sciences) and unlabeled DA (MilliporeSigma) (20 μL of ^3^H-DA per mL of 64 μM DA stock) with final DA concentrations of 6.4–0.05 μM. To evaluate dominant-negative effects of the hDAT-K619N variant on hDAT-WT function, the [^3^H]-DA uptake experiments were performed on HEK293 cells that were cotransfected with hDAT-K619N and hDAT-WT (1.5 μg of each construct) and compared with cells transfected with only hDAT-K619N or hDAT-WT (1.5 μg plus 1.5 μg empty DNA) as well as with cells transfected with 3 μg of DAT-WT as a control. Thus, the total DNA amount was kept constant at 3 μg for all conditions. Michaelis-Menten kinetics was used to fit saturation uptake data.

### In vitro amperometry recordings.

Amphetamine-induced efflux was assessed by amperometry recordings on transiently transfected HEK293 cells expressing hDAT-WT or hDAT-K619N as described previously (17).

### Live confocal microscopy of heterologous cells.

Live imaging of transiently transfected HEK293 and CAD cells was done in imaging buffer (25 mM HEPES, pH 7.4, with 130 mM NaCl, 5.4 mM KCl, 1.2 mM CaCl_2_, 1.2 mM MgSO_4_, 1 mM l-ascorbic acid, 5 mM d-glucose), and cells were reverse-transfected in 8-well Nunc LabTek II chambers (MilliporeSigma). Confocal images were obtained either on a Zeiss LSM 510 or a Zeiss LSM 780 point-scanning confocal microscope using an oil immersion 63 × 1.4 numerical aperture objective. Visualization of fluorophores and fluorescent proteins was achieved using the following laser/filter combinations: JHC 1-64 and mCherry: 543 nm helium-neon laser/560 nm long-pass filter; LysoTracker fluorophore and MFZ 9-18: 488 nm argon laser/494–565 nm bandpass filter; blue form of SlowFT: 405 nm diode laser/410–480 nm bandpass filter; red form of SlowFT: 580 nm in-tune laser/582–689 nm bandpass filter. Confocal images for all experimental series were acquired from at least 3 independent transfections.

For live cell imaging with JHC 1-64 (17, 36), cells were incubated 20 minutes with 20 nM JHC 1-64 at room temperature in imaging buffer and washed 3 times before imaging. Quantification of the mean JHC 1-64 signal was done in ImageJ. The automated “Li” threshold function was applied and mean intensity was measured. The mean intensity of all images was normalized to the WT average mean intensity for each experiment.

Imaging of mCherry-hDAT-WT or mCherry-hDAT-K619N was done in live CAD cells. Images were acquired of both mCherry alone and after 15 minutes of incubation with LysoTracker Green DND-26 (Thermo Fisher Scientific) to investigate lysosomal targeting. The mean intensity of the mCherry signal was quantified as described for JHC 1-64. The fractional overlap between mCherry- and LysoTracker-positive compartments (Manders coefficient) was calculated using the JaCoP plug-in for ImageJ (NIH).

Spatiotemporal visualization of hDAT-WT and hDAT-K619N in live CAD cells was done using N-terminal tagging with SlowFT, which is characterized (in vitro) by chromophore maturation rates of 9.8 hours for maximal blue fluorescence and 28 hours for half-maxima of the red fluorescence (38). Imaging acquisition and analysis was done as detailed in Supplemental Methods.

### Mice.

TH-Cre mice were obtained from The Jackson Laboratory, stock JAX: 8601, strain name B6.Cg-Tg(TH-Cre)1Tmd/J. These mice were backcrossed with C57BL/6N mice for at least 7 generations and maintained in a hemizygous state. All experiments were performed on adult female mice in accordance with guidelines from the Danish Animal Experimentation Inspectorate (2017-15-0201-01160)

### AAV constructs and stereotactic surgery.

Expression of hDAT-WT or hDAT-K619N in dopaminergic neurons in vivo was achieved by stereotactic injections of Cre-dependent AAV8 constructs into the midbrain of adult female TH-Cre mice (10–14 weeks). To allow detection of only the virally encoded hDAT-WT and hDAT-K619N, an HA-tag was introduced in the second extracellular loop (61). The viral vectors AAV8-hSyn-DIO-HA-hDAT-WT and AAV8-hSYN-DIO-HA-hDAT-K619N were subcloned in-house and manufactured by Vector Biolabs. The AAV8-hSYN-DIO-mCherry vector was purchased from Addgene (plasmid 50459). All 3 viral vectors had equal titers of 4.0 × 10^12^ vg/mL.

TH-Cre mice were deeply anesthetized using induction flow of 2% isoflurane and 0.5% oxygen and placed in a stereotaxic head holder (Kopf Instruments) with fitted anesthesia mask. Anesthesia was maintained using 1% to 1.5% isoflurane in 1% oxygen. The following bregma coordinates (in mm) were used to deliver unilateral or bilateral injections of 300–500 nL of AAV into the midbrain — VTA: AP –3.3, ML ± 0.5, DV –4.5 (300 nL); substantia nigra dual injections: AP –3.0, ML ± 1.2, DV –4.5 (200 nL) and AP –3.16, ML ± 1.6, DV –4.5 (300 nL).

### Synaptosomal uptake.

DA uptake experiments on crude striatal synaptosomes from TH-Cre mice bilaterally injected in VTA with AAV8-hSYN-DIO-HA-hDAT-WT, AAV8-hSYN-DIO-HA-hDAT-K619N, or AAV8-hSYN-DIO-mCherry were performed 4 to 5 weeks after injections as previously described (62), using a 2-fold dilution row (1–0.031 μM) containing a mixture of unlabeled DA and 2, 5, 6-[^3^H]-DA (PerkinElmer Life Sciences). Data were fitted to Michaelis-Menten kinetics.

### Immunohistochemistry on mice.

Mice were transcardially perfused with 4% paraformaldehyde in 0.1 M PBS (pH 7.4) 5 to 6 weeks after viral injections. Coronal sections (40 μm) from the striatum and midbrain were rinsed in PBS and incubated 30 minutes in retrieval buffer (10 mM trisodium citrate in water, pH 6.0) at 80°C. After washing, sections were blocked for 30 minutes (PBS with 5% goat serum, 1% BSA, 0.3% Triton X-100 in PBS) and incubated overnight (4°C) with primary antibodies against TH (Thermo Fisher Scientific, OPA1-04050 1:1000) and HA (Roche, 3F10 1:200). Sections were rinsed 3 times in washing buffer (0.25% BSA and 0.1% Triton X-100 in PBS) and incubated 60 minutes with secondary Alexa Fluor 488 and Alexa Fluor 568 antibodies (Abcam, ab150077 and ab175476, 1:400 in washing buffer). Sections from mice injected with AAV8-hSYN-DIO-mCherry were stained only for TH. Sections were mounted on glass coverslips (Mentzel-Gläzer, 24 × 60 mm) using ProLong Gold Antifade Mountant mounting media with DAPI (Life Technologies, P36931). Images were acquired using a Slide Scanner Axio Scan.Z1 (Zeiss) with 488 nm, 561 nm, and DAPI channel settings.

### Immunoblotting.

Whole-cell lysates from transiently transfected HEK293 cells and crude striatal synaptosomes were made as previously described (17, 31). Equal amounts of protein were incubated with loading buffer (100 mM DTT, 4× SDS loading buffer) for 1 hour at 37°C. Samples were then resolved by SDS-PAGE, and Western blotting was carried out as previously described (17) using the following antibodies: DAT (Chemicon, rat MAB369, 1:1000) or HA (Roche, rat 3F10, 1:200). After detection of HA-DAT in synaptosomal samples, the membranes were stripped in stripping buffer (62.5 mM Tris-HCl, 2% SDS, 2.5% DTT) at 50°C for 30 minutes, blocked again, and reprobed for TH using rabbit anti-TH (Thermo Fisher Scientific, OPA1-04050, 1:1000). An HRP-conjugated anti–β-actin antibody (MilliporeSigma, A3854, 1:40,000) was used as loading control. Band intensities were quantified using Fiji software. Images of full uncut gels are available in the online supplemental material.

### Amphetamine-induced rotations.

TH-Cre mice were stereotactically operated and received 2 AAV injections unilaterally (left) in lateral VTA and substantia nigra with either AAV8-hSYN-DIO-HA-hDAT-WT, AAV8-hSYN-DIO-HA-hDAT-K619N, or AAV8-hSYN-DIO-mCherry. Three weeks later, the mice were placed in open-field chambers for 1.5 hours of baseline locomotor recordings. Next, 5 mg/kg amphetamine was i.p administered, and mice were placed back in the chamber for another 1.5 hours of recording. Videos were analyzed in EthoVision to obtain baseline and amphetamine-induced locomotion and ipsilateral/contralateral rotations. Experiments and data analysis were carried out in a blinded manner.

### Drosophila genetics.

*Drosophila* strains were reared and maintained on either standard cornmeal-molasses media or on Nutri-Fly Bloomington Formulation medium (Genesee Scientific) at 25°C and under a 12-hour light/12-hour dark schedule. Transgenic flies expressing untagged versions of hDAT-WT and hDAT-K619N were used for behavioral testing, ^3^H-DA uptake, and amperometry and generated using the pBI-UASC vector (63) as previously described (19). *DAT^fmn^* (dDAT-KO), *TH-Gal4*, and *UAS-mCherry* lines were outcrossed to a *w^1118^* control line for 10 generations and selected by PCR or eye color. Likewise, flies containing the untagged UAS-hDAT-WT and UAS-hDAT-K619N transgenes were outcrossed to *DAT^fmn^* flies (in a *w^1118^* background) for 10 generations before use and maintained in this manner. Age-paired adult male flies were used for all subsequent experiments.

For visualization of the hDAT-WT and hDAT-K619N transgenes, pUASTattB-GFP-hDAT-WT and pUASTattB-GFP-hDAT-K619N constructs were custom-made by GenScript. The transgenes were inserted into the P[CaryP]attP2 site on the third chromosome using Phi31C transformation to ensure equal levels of expression. Embryo injections and selection of transformants were performed by BestGene Inc. Other fly stocks were *w^1118^* (Bloomington Drosophila Stock Center [Bl], 6326), TH-GAL4 (BI, 8848), UAS-mCherry-CAAX (Kyoto Stock Center, 109594), M[vas-int.Dm]ZH-2A (M[3xP3-RFP.attP’]ZH-22A (Bl 24481), and *DAT^fmn^* (dDAT-KO). Genotypes of the KI flies used for imaging of GFP-hDAT in dopaminergic neurons in fly brains were (WT: *w DAT^fmn^ TH-Gal4*, *UAS-mCherry-CAAX/UAS-sGFP-hDAT-WT*) and (K619N: *w DAT^fmn^ TH-Gal4*, *UAS-mCherry-CAAX/UAS-GFP-hDAT-K619N*).

### Drosophila immunostaining, imaging, and analysis.

Visualization of GFP-hDAT-WT and GFP-hDAT-K619N in brains from 2- to 5-day-old adult flies was achieved and analyzed as described in Supplemental Methods.

### Drosophila behavioral testing.

Basal activity measurements were made on male flies 3 days after eclosion placed in tubes with food for 30 hours. Activity was measured by beam breaks and analyzed using TriKinetics software.

Startle-induced negative geotactic responses were assessed in cohorts of 3-day, 23-day, and 31-day-old hDAT-WT and hDAT-K619N flies, essentially as previously described (64). Briefly, cohorts (~13–15 flies per tube) of hDAT-WT and hDAT-K619N flies were transferred to 15 cm cylinder glass tubes and habituated for 15 minutes. Negative geotactic responses were induced by 3 taps, and climbing activity was derived as the percentage of flies passing a 6 cm line after 5 seconds in young (3-day-old) flies or after 10 seconds in older flies. Each cohort was tested in 15 trials with a 1-minute intertrial period. At least 13 cohorts of each genotype were assessed at any time point, and the mean climbing activity was compared for hDAT-WT and hDAT-K619N.

### Drosophila ^3^H-DA uptake and amperometry assays.

DA uptake and amperometric recordings of amphetamine-induced efflux in isolated *Drosophila* brains were carried out as previously described (23).

### Statistics.

GraphPad Prism 8.0 software was used for data fitting and statistical analysis. Statistical methods for all comparisons are described in figure legends. Unless otherwise stated, a 1-sample 2-tailed *t* test was applied to examine relative differences, i.e., for normalized data; absolute measures were compared using either a paired or unpaired *t* test (depending on the experimental design) for normally distributed data or Mann-Whitney test for data that failed normality tests. One-way or 2-way ANOVA with a Holm-Šídák posttest were used for multiple comparisons. *P* values of less than 0.05 were considered significant.

### Study approval.

Patients 1 and 2 were included from previously described patient samples (17, 33). Written informed consent was obtained from patient 1 for all further investigations, including the use of videos in this manuscript. The iPSYCH study sample was approved by the Danish Data Protection Agency. Informed consent is not required by law for register-based research in Denmark (47). Experimental procedures on animals adhered to the European guidelines for the care and use of laboratory animals, Directive 2010/63/EU, and were approved by the Danish Animal Experimentation Inspectorate (2017-15-0201-01160 and 2017-15-0201-01177). All efforts were made to minimize pain and discomfort as well as the number of animals used in each experiment.

## Author contributions

FH and UG conceptualized the study. LEH identified, examined, and included patient 1 and coordinated his clinical procedures. Information on patient 2 was provided by AG. FH, KLJ, ST, NVA, AS, JA, LPP, AHR, HM, KE, TS, VKL, and MR conducted the in vivo, ex vivo, and in vitro experiments. AL and MNL provided and quantified DAT SPECT scans. HM and AG generated the untagged drosophila KI lines; OK and VKL generated the GFP-tagged drosophila lines; TRC and ATS designed and cloned AAV constructs. LBM and TS carried out sequencing of patient 1 and his parents. TW provided the iPSYCH information on DAT-K619N from exome data. AHN provided JHC 1-64 and MFZ 9-18, synthesized in the Medicinal Chemistry Section, NIDA-Intramural Research Program. FH, KLJ, and UG prepared data and performed the formal data analyses. FH and UG provided funding, prepared figures, and wrote the manuscript. All authors contributed to the editing and review of the manuscript.

## Supplementary Material

Supplemental data

Supplemental video 1

Supplemental video 2

Supplemental video 3

## Figures and Tables

**Table 1:**
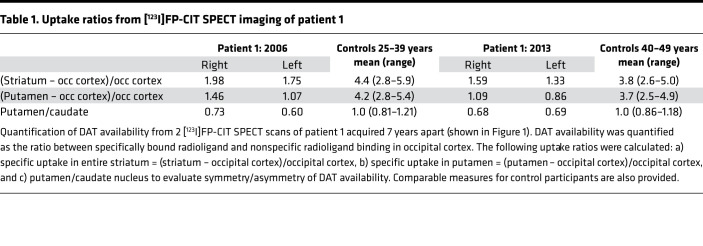
Uptake ratios from [^123^I]FP-CIT SPECT imaging of patient 1

**Table 2:**
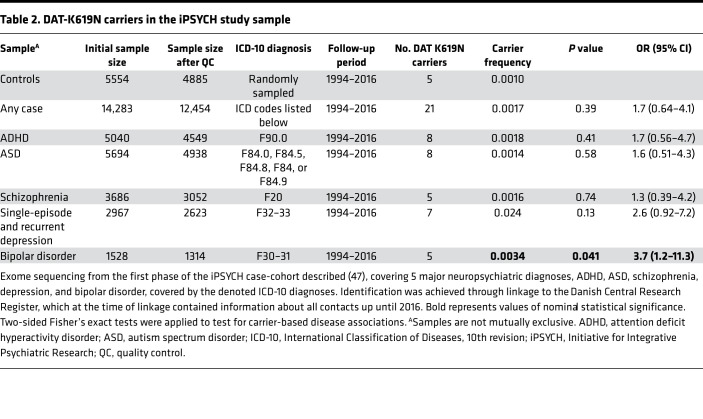
DAT-K619N carriers in the iPSYCH study sample

## Version 1 08/10/2021

In-Press Preview

## Version 2 09/22/2021

Electronic publication
